# Effect of dopamine on TGF-β2 secretion by human retinal pigment epithelial cells and the underlying mechanism

**DOI:** 10.1371/journal.pone.0335526

**Published:** 2025-11-04

**Authors:** Jing Fan, Qiujin Zhang, Liu Zheng, Binbin Yang, He Jin, Tingyu Meng, Zhixiang Ding

**Affiliations:** 1 The 988 Hospital of the Joint Logistic Support Force of the Chinese People’s Liberation Army, Zhengzhou City, Henan Province, China; 2 Guilin Medical University, Qixing District, Guilin City, Guilin, China; 3 The Affiliated Hospital of Guilin Medical College, Guilin City, Guangxi Zhuang Autonomous Region, China; 4 Taihe Hospital, Maojian District, Shiyan City, Hubei Province, China; 5 Department of Ophthalmology, The Affiliated Hospital of Guilin Medical College, Guilin City, Guangxi Zhuang Autonomous Region, China; Apeejay Stya University, INDIA

## Abstract

**Background:**

Dopamine is known to play a role in the development of myopia. In this study, we investigated the effect of dopamine (DA) on the secretion of transforming growth factor-β2 (TGF-β2) in human retinal pigment epithelial (RPE) cells. Additionally, we examined the effects of SCH23390 (D1 receptor antagonist) and sulpiride (D2 receptor antagonist) on TGF-β2 levels in ARPE-19 cells to elucidate the underlying mechanism of DA in myopia development.

**Methods:**

Human RPE cells were cultured with or without different concentrations of DA, SCH23390, or sulpiride. Cell proliferation was examined using the CCK-8 assay. Cell migration was assessed by performing a cell scratch (wound healing) assay. The expression of TGF-β2 was evaluated at the mRNA and protein levels by real-time PCR and western blotting, respectively. Furthermore, the secretion of TGF-β2 into the ARPE-19 cell supernatant was quantified using ELISA.

**Results:**

Treatment with 20 μg/mL of DA significantly increased (*P* < 0.05) the mRNA and protein expression levels of DRD1, DRD2, YAP, and TEAD and decreased TGF-β2 levels compared with that in control group. However, treatment with 24 μg/mL of SCH23390 for 24 h significantly decreased (*P* < 0.05) *DRD1* and *TGF-β2* mRNA expression and increased *DRD2*, *YAP*, and *TEAD* mRNA expression in ARPE-19 cells compared with that in the control group. Additionally, the protein expression levels of TGF-β2, YAP, TEAD, and SMAD7 were consistent with the mRNA levels. Notably, treatment with sulpiride (14 μg/mL) increased (*P* < 0.05) *DRD1* and *TGF-β2* expression and decreased *DRD2*, *YAP*, and *TEAD* expression in the cells at both the mRNA and protein levels compared with that in the control group.

**Conclusions:**

Our findings suggest that dopamine suppresses TGF-β2 secretion in human retinal pigment epithelial cells primarily through activation of D2 receptors, which appears to involve the YAP-TGF-β-Smad signaling pathway. This regulatory mechanism may contribute to the control of scleral remodeling and thus influence the development of myopia.

## 1. Background

The prevalence of myopia has increased dramatically in East Asia and globally over the past decades, posing a significant public health challenge. Epidemiological and animal studies have consistently demonstrated an inverse correlation between time spent outdoors (under high-intensity light) and the risk of developing myopia [1–6]. Lifestyle changes, including increased educational pressure and reduced outdoor activities, are believed to contribute to the rising myopia rates in East Asian urban populations [7]. Understanding the biological mechanisms underlying this association is therefore critical for developing effective interventional strategies.

According to the theory of retinal local regulation, the retina produces first-order signaling factors (such as dopamine) when light defocus or form deprivation occurs. Importantly, these signaling factors further act on RPE cells to produce secondary signaling factors (such as MMP-2 and TGF-β2), which ultimately play an important regulatory role in the development of the eye [8]. Moreover, it is widely believed that DA is a myopia development “stop” signal [9].

Retinal pigment epithelial (RPE) cells play a pivotal role in this process by relaying retinal growth signals to the choroid and sclera. Although DA is primarily released by specific amacrine cells, it exerts its effects by activating D1 and D2 family dopamine receptors distributed throughout the retina [10]. DA regulates several functions, including the development of retinal, visual, and refractive systems [10–12]. Importantly, the relationship between DA and eye development was first published in 1989 [13]. Research findings indicate that DA antagonizes the development of myopia in the retina [14]. The “light-dopamine” hypothesis further proposes that bright light exposure elevates retinal DA levels, [2], thereby protecting against myopia [15–17],Consequently, enhancing DA signaling presents a potential therapeutic strategy for myopia control.

The ARPE-19 cell line, a well-established in vitro model of human RPE, is commonly used to study refractive development [18]. DA signals through G protein-coupled receptors, classified into D1-like (D1, D5) and D2-like (D2, D3, D4) families, which differentially modulate intracellular cAMP levels [19]. D1 receptor activation stimulates adenylyl cyclase, increasing cAMP, whereas D2 receptor activation inhibits it, reducing cAMP levels. This opposing signaling underscores the complexity of DA’s actions. Previous studies suggest species-specific roles: D2 receptor antagonism disrupted emmetropization in chickens [20]. while D1-like mechanisms mediated DA’s protective effects in mice [21].Furthermore, recent evidence indicates that dopaminergic drugs may act directly through retinal D1 and D2 receptors rather than indirectly altering DA levels [22].although the specific role of D1 receptors in ocular growth remains less clear.

In abnormal visual conditions, altered expression of retinal neurotransmitters and cytokines leads to scleral remodeling. RPE cells are a major source of TGF-β, a critical cytokine in myopia development. Specifically, TGF-β2 secreted by RPE cells is a key mediator of scleral extracellular matrix changes [23,24].Clinical studies have shown elevated TGF-β2 levels in the aqueous humor of highly myopic patients, correlating positively with axial length [25].

Notably, the Hippo pathway controls cell proliferation, tissue homeostasis, and organ size through Yes-associated protein (YAP) and its collateral homologs [26]. Its downstream effectors, Yes-associated protein (YAP) and transcriptional enhancer factor TEA domain (TEAD) [27,28],shuttle between the cytoplasm and nucleus to control gene expression. Notably, YAP can interact with Smad proteins, key transducers of TGF-β signaling [29,30]. Specifically, YAP1 binds to Smad7 and enhances its inhibition of TGF-β receptor activity [31], suggesting a potential cross-talk between Hippo/YAP and TGF-β pathways in regulating RPE function.

Despite these advances, the mechanism by which DA influences TGF-β2 secretion in human RPE cells and its potential regulation through the YAP-TGF-β-Smad axis remains unexplored. Therefore, this study aimed to investigate the effect of dopamine on TGF-β2 expression in ARPE-19 cells and to elucidate the involvement of specific dopamine receptors (using antagonists SCH23390 and sulpiride) and the YAP-TGF-β-Smad signaling pathway in this process.

## 2. Methods

### 2.1 Cell culture and treatment

ARPE-19 cells (Shanghai Cell Bank) were routinely cultured in DMEM/F12 (1:1) medium containing 10% fetal bovine serum and penicillin-streptomycin solution in a CO_2_ incubator at 37°C. The culture medium was changed every 2–3 days. At 80% confluence, the cells were digested with Trypsin-EDTA solution in T25 culture flasks at a passage ratio of 1:2–1:3 and used for subsequent experiments.

DA hydrochloride (H8502; Sigma-Aldrich, St. Louis, MO, USA) was dissolved in ddH_2_O at a concentration of 100 mg/mL to prepare a stock solution. SCH23390 hydrochloride (GLPBIO) and sulpiride (GLPBIO) were dissolved in dimethyl sulfoxide (DMSO; Sigma-Aldrich) to a concentration of 2000 μg/mL, further diluted to the working concentration using DMEM/F12, and immediately frozen at –20°C or with an equal amount of reagent in the dark.

### 2.2 Cell counting kit-8 (CCK-8) assay

ARPE-19 cells were pre-cultured for 8–12 h (37°C, 5% CO_2_) with different concentrations of DA (10, 20, 40, and 80 μg/mL), SCH23390 (12, 24, 48, and 92 μg/mL), or sulpiride (7, 14, 28, and 56 μg/mL). Thereafter, CCK-8 solution (10 μL) was added to each well of the culture plates, followed by incubation at 37°C in 5% CO_2_ for 1 h. Finally, absorbance was measured at 450 nm using a PowerWave XS Microplate Spectrophotometer (Bio-Tek, USA). All experiments were repeated thrice independently. Data are expressed as the mean ± standard deviation of the percentage of living cells.

Cell activity was calculated using the following formula:


$$\text{Cell}\;\;\text{viability}(\text{1}\text{0}\text{0}\%)=\frac{A_{experiment\;group}\;-\;A_{blank\;group}}{A_{control\;group}\;-\;A_{blank\;group}}\times \text{1}\text{0}\text{0}\%$$

where A_(experiment group)_ denotes the absorbance of wells with cells, CCK8 solution, and drug solution; A_(blank group)_ denotes the absorbance of wells with culture medium and CCK8 solution without cells; and A_(control group)_ denotes the absorbance of wells with cells, CCK8 solution, but no drug solution.

### 2.3 Cellular scratch test

A marker was used to draw horizontal lines evenly with a ruler under the wells of a 6-well plate and across the wells at intervals of 0.5–1.0 cm, with each well having at least five lines passing through it. Briefly, ARPE cells (2.5 × 10^4^) were seeded in the wells and incubated for indicated durations. After adherence, ARPE-19 cell monolayers were scratched with a 200-μL micropipette tip perpendicular to the horizontal line and washed with phosphate-buffered saline three times to remove the scratched cells. Thereafter, the scratched cells were photographed after adding the culture medium for 0 h and DA (10 or 20 μg/mL), SCH23390 (12 or 24 μg/mL), or sulpiride (7 or 14 μg/mL) for 12 h. Cell migration ability was expressed as the cell mobility (%) and was calculated using the following formula:


$$\text{ Cell mobility}(\%)=\frac{\text{0h scratch width}-\text{12h scratch width}}{\text{0h scratch width }}\times \text{100}\%$$

### 2.4 RT-PCR

Total RNA was isolated from cultured cells using Trizol reagent (Invitrogen, Thermo Fisher Scientific, USA, catalogue no.10296–010), according to the manufacturer’s instructions. The concentration and purity of the extracted RNA were measured using a NanoDrop spectrophotometer. Thereafter, 1 μg of total RNA was reverse-transcribed to generate cDNA using the FastKing RT Kit (With gDNase) (Tiangen Biochemical Technology, China, catalogue no. KR118–01). RT-PCR was performed on an ABI 7500 Real-Time PCR System (Applied Biosystems, USA) using the MonAmp™ 2 × Taq Mix (+Dye) (Monad Biotech, China, catalogue no. [MP05401S]). The primer stock solution was 10 μM.The 20 μL reaction mixture contained 10 μL of MonAmp™ 2 × Taq Mix (+Dye), 1 μL of each forward and reverse primer, 1 μL of cDNA template, and nuclease-free water up to 20 μL,resulting in a final concentration of 0.5 μM for each primer. The PCR amplification program was as follows: pre-denaturation at 94°C for 3 min; 20–35 cycles of denaturation at 94°C for 15 s, annealing at 54°C for 15 s, and extension at 72°C for 15 s; and a final extension at 72°C for 3 min. The PCR products were separated via SDS-PAGE gel electrophoresis and photographed under ultraviolet irradiation.The primer pairs used for RT-PCR analysis are listed in Table 1.

**Table 1:** Primer sequences used to amplify the genes of interest in this study.

Gene	Primer sequences
*DRD1*	Forward: 5′- GGGGCTTTGAGAGACGAC -3′
	Reverse: 5′- CATTTCGGGGCTGTTGCTTT -3′
*DRD2*	Forward: 5′-ATACGCGCTACAGCTCCAAG-3′
	Reverse: 5′-CAGGGTGACAATGAAGGGCA-3′
*TGF-β2*	Forward: 5′-CAGAGTGCCTGAACAACGGA-3′
	Reverse: 5′-GGTACAAAAGTGCAGCAGGG-3′
*YAP*	Forward: 5′-AACACTGGAGCAGGATGGTG-3′
	Reverse: 5′-GGGTTCGAGGGACACTGTAG-3′
*TEAD*	Forward: 5′-GGGTTCGAGGGACACTGTAG-3′
	Reverse: 5′-GTTGTGCTCCGTGTTCACTATTT-3′
*GAPDH*	Forward: 5′-GGAGTCCACTGGCGTCTTCA-3′
	Reverse: 5′-GTCATGAGTCCTTCCACGATACC-3′

### 2.5 Western blotting

Proteins were extracted from treated cells using histiocyte lysate and benzyl sulfonyl fluoride (PMSF) (100:1), followed by the determination of their concentrations using a BCA protein concentration assay kit (Beyotime, Shanghai, China). Thereafter, the proteins were separated using 10% sodium dodecyl sulfate (SDS)-polyacrylamide gel electrophoresis and transferred to a polyvinylidene fluoride (PVDF) membrane (Millipore, USA). After blocking with 5% skim milk at 25°C for 2 h, the membranes were incubated overnight at 4°C with the following primary antibodies: rabbit monoclonal anti-YAP (ab205270) and rabbit monoclonal anti-TEAD antibodies (ab133533) from Abcam (USA); rabbit polyclonal anti-TGF-β antibodies (GTX132546-S) from GeneTex (USA); and anti-GAPDH antibodies (L3012-0.1) from Proteintech (USA). The membranes were washed with tris buffered saline with tween (TBST) and incubated with goat anti-rabbit IgG secondary antibody (Cell Signaling Technology, cat:7074) coupled with horseradish peroxidase at room temperature for 2 h. Protein bands were visualized using an ECL Test Kit (Millipore) and quantified using Image Lab software (Bio-Rad, Hercules, CA). Notably, the expression levels of the proteins were normalized to that of GAPDH.

### 2.6 Enzyme-linked immunosorbent assay (ELISA)

Cells were inoculated into 6-well plates at a density of 2.5 × 10^4^ cells/well. Thereafter, the cell culture supernatant was collected and centrifuged at 4000 rpm for 20 min to remove cell particles and polymers. The supernatants were stored at ˗20°C until further use. Finally, the levels of TGF-β2 in the supernatants were measured using a TGF-β2 ELISA kit (Ruixin Biotech, RX104767H).

### 2.7 Statistical analysis

Quantitative data are expressed as the mean ± standard deviation. Significant differences between groups were determined using one-way analysis of variance (ANOVA), followed by Bonferroni’s multiple comparisons test. The Bartlett test was used to determine the homogeneity of the variance obtained. Statistical significance was set at *P* < 0.05 (* P < 0.05, ** P < 0.001).

### 2.8 Ethics approval and consent to participate

This study used commercially available cell lines and did not involve any human or animal subjects. Therefore, ethical approval and consent to participate were not applicable.

## 3 Results

### 3.1 DA promotes the proliferation of ARPE-19 cells

**Fig 1:**
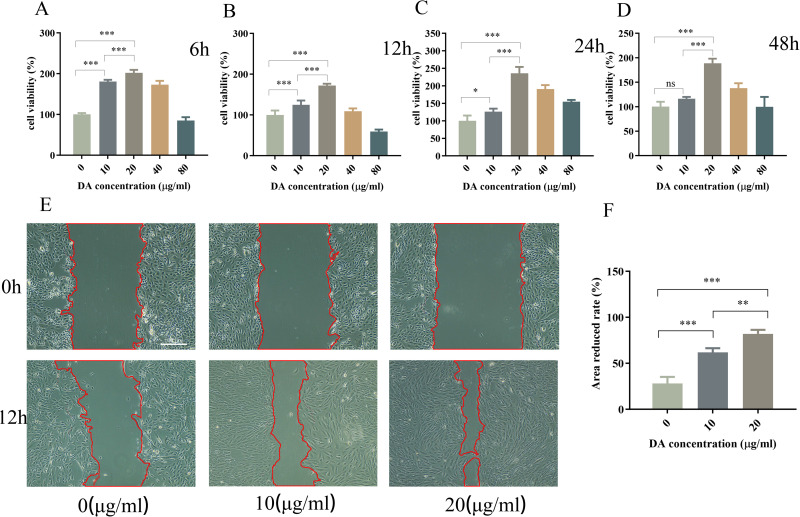
Viability and migration ability of ARPE-19 cells after dopamine (DA) administration. **(A–D)** ARPE-19 cell viability after treatment with different concentrations of DA (10, 20, 40, and 80 μg/mL) for 6, 12, 24, or 48 h. The control group was kept under the same conditions without adding DA. **(E)** Transwell migration images of ARPE-19 cells treated with 0, 10, or 20 μg/mL DA for 0 and 12 h, and **(F)** quantitative results. Scale bars: 100 μm. Data are reported as the means ± SD, n = 3. *p < 0.05, **p < 0.01, ***p < 0.001.

CCK-8 assay showed that treatment with 20 μg/mL of DA for 6, 12, 24, and 48 h significantly increased (*P* < 0.05) the viability of ARPE-19 cells compared with that in the control group (Figs 1A–1D), peaking at 24 h of treatment (*P* < 0.05; Fig 1C).

### 3.2 Effect of DA on the migration ability of ARPE-19 cells

Cell migration assay showed that treatment with 10 and 20 μg/mL of DA promoted cell migration ability (Figs 1E–1F), with DA dose of 20 μg/mL showing the best performance compared with that the in control group (*P* < 0.05; Fig 1F). Therefore, 20 μg/mL of DA was selected for subsequent experiments.

### 3.3 Effect of DA on *DRD1,DRD2,YAP*, *TGF-β2*, and *TEAD* mRNA expression in ARPE-19 cells

**Fig 2:**
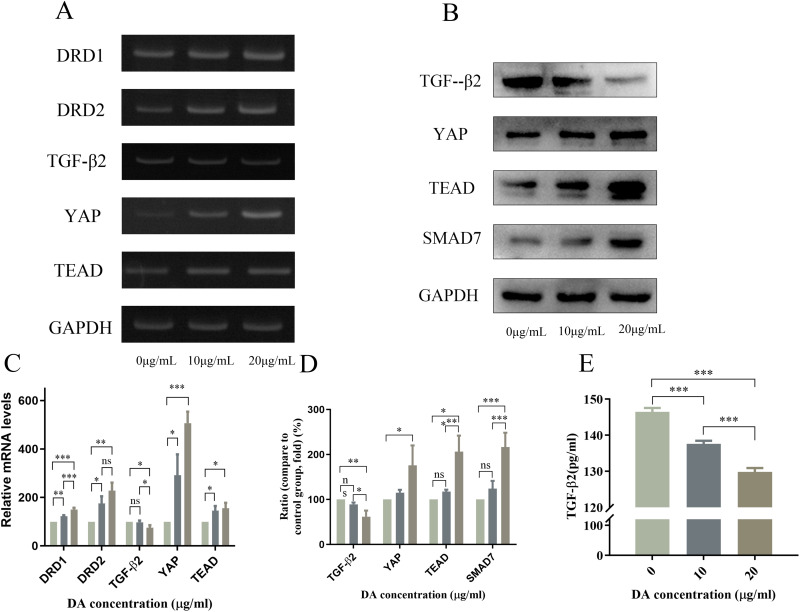
Effects of dopamine (DA) on mRNA and protein expression of factors in the YAP-TGF-β-Smad signaling pathway in ARPE-19 cells. **(A)** RT-PCR was used to detect the mRNA expression of *DRD1, DRD2, YAP, TEAD, and TGF-β2* in ARPE-19 cells, **(B)** Western blotting was used to detect the protein expression of *SMAD7, YAP, TEAD, and TGF-β2* in ARPE-19 cells, **(C)** Quantitative analysis of *DRD1, DRD2, YAP, TEAD and TGF-β2* mRNA expression levels in ARPE-19 cells. **(D)** quantitative results of protein expression of *SMAD7, YAP, TEAD, and TGF-β2* in ARPE-19 cells. **(E)** Protein expression of *TGF-β2* in the supernatant of ARPE-19 cell cultures, determined using ELISA. Data are reported as the means ± SD, n = 3. *p < 0.05, **p < 0.01, ***p < 0.001.

To investigate whether DA acts through the YAP-TGF-β-Smad signaling pathway, we examined the mRNA expression of *DRD1*, *DRD2*, *YAP*, and *TEAD* in DA-treated ARPE-19 cells. RT-PCR showed that the mRNA expression levels of *DRD1, DRD2, YAP* and *TEAD* increased in ARPE-19 cells (P < 0.01).Notably, DA treatment caused a dose-dependent decrease (*P* < 0.01) in *TGF-β2* mRNA expression compared with that in the control group (Figs 2A and 2C).

### 3.4 Effect of DA on YAP, TGF-β2, TEAD, and SMAD7 protein expression in ARPE-19 cells

Western blotting revealed that DA treatment (20 μg/mL) significantly increased (*P* < 0.05) YAP, TEAD, and SMAD7 protein levels and decreased (*P* < 0.01) TGF-β2 protein expression in ARPE-19 cells compared with that in the control group (Figs 2B and 2D). Similarly, TGF-β2 expression was significantly downregulated (*P* < 0.001) ARPE-19 cell supernatant following DA treatment (20 μg/mL) compared with that in the control group (Fig 2E). Overall, these results indicate that DA treatment may reduce TGF-β2 expression by promoting YAP and SMAD7 levels and binding to ARPE-19 cells, suggesting that the YAP-TGF-β-Smad signaling pathway may be involved in the DA-mediated regulation of TGF-β2 expression.

### 3.5 SCH23390 affects the proliferative capacity of ARPE-19 cells

**Fig 3:**
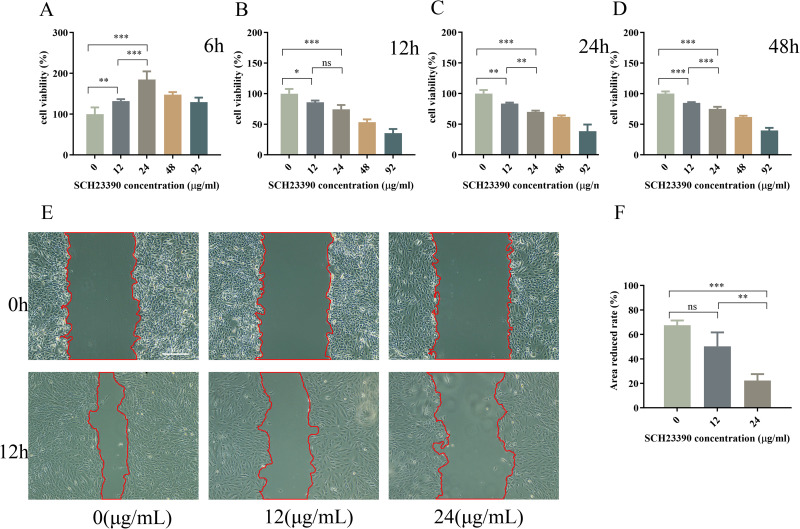
ARPE-19 cell viability and migration ability after SCH23390 administration. **(A–D)** ARPE-19 cell viability after treatment with different concentrations of SCH23390 (12, 24, 48, or 92 μg/mL) for 6, 12, 24, or 48 h. The control group was treated similarly without the addition of SCH23390. **(E)** Transwell migration images of ARPE-19 cells treated with 0, 12, or 24 μg/mL SCH23390 for 0 and 12 h, and **(F)** the quantitative results. Scale bars: 100 μm. Data are reported as the means ± SD, n = 3. *p < 0.05, **p < 0.01, ***p < 0.001.

To investigate whether DA signaling is involved in refractive development, we examined the effects of D1R and D2R inhibitors on ARPE-19 cells. Although SCH23390 treatment was not significant at 6 h (Fig 3A), ARPE-19 cell viability was significantly inhibited (*P* < 0.05) after 12 h of treatment with 24 μg/mL of SCH23390 compared with that in the control group (Figs 3C and 3D). Thereafter, 24 μg/mL of SCH23390 was selected as the optimal drug concentration based on its half-maximal inhibitory concentration (IC_50_).

### 3.6 SCH23390 affects the migration ability of ARPE-19 cells

Cell scratch assay showed that SCH23390 treatment (24 μg/mL) significantly decreased (*P* < 0.001) the migration ability of ARPE cells compared with in the control group (Fig 3E).

### 3.7 SCH23390 affects the expression of DRD1, DRD2 YAP, TGF-β and TEAD mRNA in ARPE-19 cells

**Fig 4:**
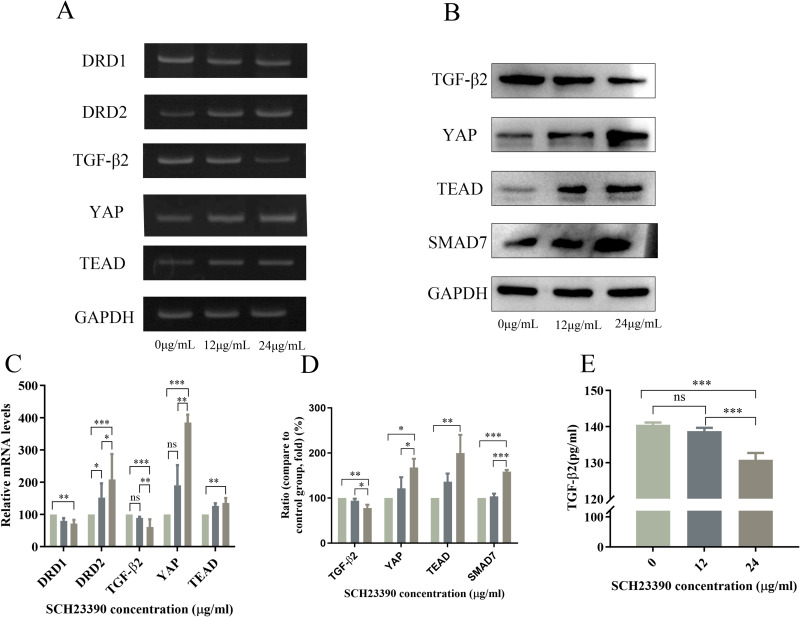
Effects of SCH23390 on mRNA and protein expression of factors in the YAP-TGF-β-Smad signaling pathway in ARPE-19 cells. **(A)** RT-PCR was used to detect the mRNA expression of *DRD1, DRD2, YAP, TEAD, and TGF-β2* in ARPE-19 cells, **(B)** Western blotting was used to detect the protein expression of *SMAD7, YAP, TEAD, and TGF-β2* in ARPE-19 cells, **(C)** Quantitative analysis of *DRD1, DRD2, YAP, TEAD and TGF-β2* mRNA expression levels in ARPE-19 cells. **(D)** quantitative results of protein expression of *SMAD7, YAP, TEAD, and TGF-β2* in ARPE-19 cells. **(E)** Protein expression of *TGF-β2* in the supernatant of ARPE-19 cell cultures, determined using ELISA. Data are reported as the means ± SD, n = 3. *p < 0.05, **p < 0.01, ***p < 0.001.

To investigate whether DA receptors are involved in the development of refractive development, we examined *DRD1, DRD2,YAP*, *TGF-*β*2* and *TEAD* mRNA expression in ARPE-19 cells treated with 24 μg/mL of SCH23390 for 24 h using RT-PCR. SCH23390 treatment caused a significant dose-dependent decrease (*P* < 0.01) in *DRD1* and *TGF-β2* mRNA expression and a dose-dependent increase (*P* < 0.01) in *DRD2*, *YAP*, and *TEAD* mRNA expression compared with that in the control group (Figs 4A and 4C).

### 3.8 SCH23390 affects the protein expression of YAP, TGF-β2, TEAD, and SMAD7 in ARPE-19 cells

Western blotting showed that SCH23390 treatment (24 μg/mL) for 24 h significantly increased (*P* < 0.05) YAP, TEAD, and SMAD7 protein levels and significantly decreased (*P* < 0.01) TGF-β2 protein expression in RPE cells compared with that in the control group (Figs 4B and 4D). Similarly, ELISA showed a significantly decrease (*P* < 0.001) in TGF-β2 expression in ARPE-19 cell supernatant following treatment with SCH23390 (24 μg/mL) compared with that in the control group (Fig 4E). Collectively, these results suggest that SCH23390 treatment, which antagonizes D1R, led to reduced TGF-β2 expression and altered levels of YAP and SMAD7, suggesting a potential interaction within the YAP-TGF-β-Smad pathway.

### 3.9 Effect of sulpiride on the proliferation of ARPE-19 cells

**Fig 5:**
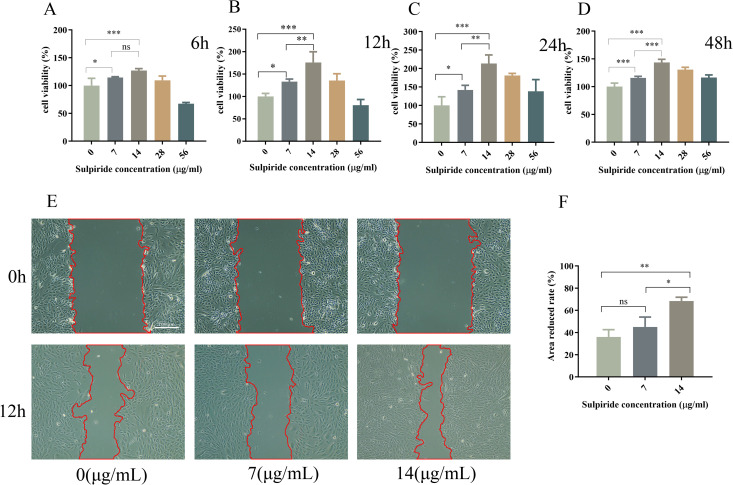
ARPE-19 cell viability and migration ability after sulpiride administration. **(A–D)** ARPE-19 cell viability after treatment with different concentrations of sulpiride (7, 14, 28, or 56 μg/mL) for 6, 12, 24, or 48 h. The control group was maintained under the same conditions without the addition of sulpiride. **(E)** Transwell migration images of ARPE-19 cells treated with 0, 7, or 14 μg/mL sulpiride for 0 and 12 h, and **(F)** the quantitative results. Scale bars: 100 μm. Data are reported as the means ± SD, n = 3. *p < 0.05, **p < 0.01, *** *p* < 0.001.

Based on CCK-8 assay, 14 μg/mL of sulpiride resulted in optimal cell viability at 6, 12, 24, and 48 h. Notably, sulpiride treatment (14 μg/mL) significantly increased (*P* < 0.05) the viability of ARPE-19 cells compared with that in the control group (Figs 5A–5D).

### 3.10 Effect of sulpiride on the migration ability of ARPE-19 cells

Cell scratch assay showed a significant increase (*P* < 0.05) in the migration ability of ARPE-19 cells following treatment with 14 μg/mL of sulpiride for 12 h (Figs 5E and 5F). Based on these results, 14 μg/mL of sulpiride was selected for subsequent experiment.

### 3.11 Sulpiride affects the mRNA expressions of DRD1, DRD2 *YAP*, *TGF-β2*, and *TEAD* in ARPE-19 cells

**Fig 6:**
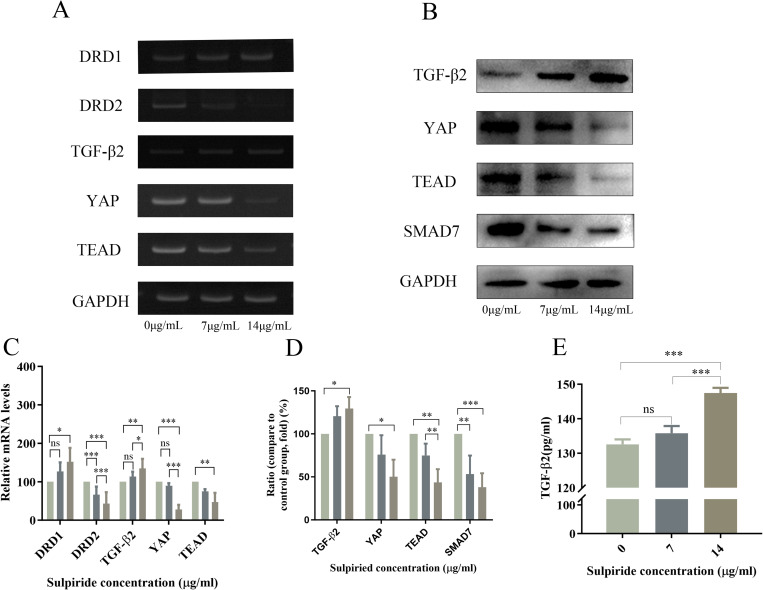
Effects of sulpiride on mRNA and protein expression of factors in the YAP-TGF-β-Smad signaling pathway in ARPE-19 cells. **(A)** RT-PCR was used to detect the mRNA expression of *DRD1, DRD2, YAP, TEAD, and TGF-β2* in ARPE-19 cells, **(B)** Western blotting was used to detect the protein expression of *SMAD7, YAP, TEAD, and TGF-β2* in ARPE-19 cells, **(C)** Quantitative analysis of *DRD1, DRD2, YAP, TEAD and TGF-β2* mRNA expression levels in ARPE-19 cells. **(D)** quantitative results of protein expression of *SMAD7, YAP, TEAD, and TGF-β2* in ARPE-19 cells. **(E)** Protein expression of TGF-β2 in the supernatant of ARPE-19 cell cultures, determined using ELISA. Data are reported as the means ± SD, n = 3. *p < 0.05, **p < 0.01, ***p < 0.001.

RT-PCR showed that sulpiride treatment (14 μg/mL) for 24h caused a significant dose-dependent increase (*P *< 0.05) in *DRD1* and *TGF-β2* mRNA expression levels and dose-dependent decrease (*P* < 0.01) in *DRD1 2*, *YAP*, and *TEAD* mRNA expression in ARPE-19 cells compared with that in the control group (Figs 6A and 6C).

### 3.12 Sulpiride affects the expression of YAP, TGF-β2, TEAD, and SMAD7 proteins in ARPE-19 cells

Western blotting showed that sulpiride treatment (14 μg/mL) for 24 h significantly decreased (*P* < 0.05) YAP, TEAD, and SMAD7 protein levels and significantly increased (*P *< 0.05) TGF-β2 protein expression compared with that in the control group (Figs 6B and 6D). Similarly, ELISA showed a significant increase (*P* < 0.001) TGF-β2 expression in ARPE-19 cell supernatant following sulpiride treatment (14 μg/mL) compared with that in the control group (Fig 6E). Overall, these results suggest that sulpiride inhibits the binding of DA to D2R, decreasing the expression of YAP1 and SMAD7 and increasing the expression of TGF-β2 in ARPE-19 cells. Conclusively, sulpiride may affect TGF-β2 expression through the YAP-TGF-β-Smad signaling pathway.

## 4. Discussion

The increasing global prevalence of myopia necessitates a deeper understanding of its underlying molecular mechanisms. In this study, we demonstrate that dopamine modulates TGF-β2 secretion in human RPE cells via the YAP-TGF-β-Smad signaling pathway, primarily through D2 receptor activation, providing novel insights into the regulatory network controlling scleral remodeling and ocular growth.

### DA-mediated inhibition of TGF-β2 secretion

Our findings indicate that DA significantly suppresses TGF-β2 expression at both mRNA and protein levels in ARPE-19 cells. This result aligns with the well-established role of dopamine as a “stop signal” in myopia progression [14,19]. Previous studies have demonstrated that increased retinal dopamine activity inhibits axial elongation in various animal models of myopia [13,21]. However, the exact mechanism through which DA influences downstream signaling molecules in RPE cells remains poorly understood. Our study extends these observations by identifying TGF-β2 as a key downstream target of DA signaling in human RPE cells, providing a mechanistic link between retinal dopamine activity and scleral remodeling processes.

### Differential roles of dopamine receptors in TGF-β2 regulation

A significant finding of our study is the differential involvement of dopamine receptor subtypes in regulating TGF-β2 expression. While both D1 and D2 receptors responded to DA stimulation, our pharmacological approach using selective antagonists revealed that the anti-myopic effects of DA are primarily mediated through D2 receptors. SCH23390 (D1 antagonist) treatment decreased DRD1 expression but unexpectedly increased DRD2, YAP, and TEAD expression, ultimately reducing TGF-β2 levels. In contrast, sulpiride (D2 antagonist) increased DRD1 and TGF-β2 expression while decreasing DRD2, YAP, and TEAD expression. These results suggest that D2 receptor activation is primarily responsible for the inhibitory effect on TGF-β2 secretion. This finding corroborates with Huang et al.[21], who demonstrated that D2 receptor activation protects against form-deprivation myopia in mice. However, our results appear to contradict some studies suggesting D1 receptor involvement in myopia protection [15], highlighting the complexity of dopaminergic signaling in ocular growth regulation. These discrepancies might be attributed to species differences, experimental models, or specific signaling contexts.

### Involvement of the YAP-TGF-β-Smad pathway

We provide compelling evidence that the YAP-TGF-β-Smad pathway mediates DA’s effects on TGF-β2 regulation in RPE cells. DA treatment significantly increased the expression of YAP, TEAD, and SMAD7 while decreasing TGF-β2 expression. This finding is particularly significant as it identifies a novel signaling pathway through which dopamine may influence scleral remodeling. The Hippo/YAP pathway has recently emerged as a critical regulator of organ size and tissue homeostasis [26,28], but its role in myopia development remains largely unexplored. Our observation that YAP and TEAD expression patterns correlate with TGF-β2 levels suggests their involvement in DA signaling. This finding is supported by previous reports demonstrating interactions between YAP and Smad proteins in other systems [29,30]. Specifically, YAP1 can bind to Smad7 and enhance its inhibitory effect on TGF-β receptor activity [31], providing a potential mechanism for our observed results. While the precise molecular interactions require further elucidation, our study offers the first evidence connecting dopaminergic signaling with the YAP-TGF-β-Smad axis in RPE cells.

### Functional implications of DA-induced migration despite TGF-β2 reduction

An intriguing observation from our study is that DA enhanced ARPE-19 cell migration despite reducing TGF-β2 expression, which typically promotes migration in many cell types. This apparent paradox suggests that DA might influence RPE cell behavior through multiple mechanisms, possibly by activating additional signaling pathways that override or modify TGF-β2’s effects. Alternatively, the net effect on migration may reflect a balance between various pro-migratory and anti-migratory signals regulated by DA. This complex regulation emphasizes the need for further investigation into the diverse functions of DA in ocular cell biology.

### Study limitations and future perspectives

We acknowledge several limitations in our study. First, we used ARPE-19 cells as an in vitro model, which may not fully recapitulate the complexity of native RPE physiology. Future studies using primary RPE cells or in vivo models would validate these findings. Second, our experimental approach analyzed different targets at different levels (dopamine receptors at mRNA level and SMAD7 at protein level) due to technical challenges associated with reliably detecting GPCR proteins via Western blotting and the functional relevance of measuring SMAD7 protein levels. Future studies employing techniques like flow cytometry for receptor surface expression or RNA-seq for comprehensive transcriptomic analysis could provide additional layers of evidence. Finally, while we demonstrated associations between DA signaling and the YAP-TGF-β-Smad pathway, direct molecular interactions require further investigation.

## Conclusion

In conclusion, our study provides evidence that dopamine inhibits TGF-β2 secretion in human RPE cells primarily through D2 receptor activation and involves the YAP-TGF-β-Smad signaling pathway. These findings establish a novel connection between dopaminergic signaling and mechanotransduction pathways in regulating ocular growth, offering potential therapeutic targets for myopia intervention. Future research should focus on validating these mechanisms in animal models and exploring translational applications for myopia prevention and treatment.

## Supporting information

S1 Fig Viability and migration ability of ARPE-19 cells after dopamine (DA) administration. (A–D) ARPE-19 cell viability after treatment with different concentrations of DA (10, 20, 40, and 80 μg/mL) for 6, 12, 24, or 48 h. The control group was kept under the same conditions without adding DA. (E) Transwell migration images of ARPE-19 cells treated with 0, 10, or 20 μg/mL DA for 0 and 12 h, and (F) quantitative results. Scale bars: 100 μm. Data are reported as the means ± SD, n = 3. *p < 0.05, **p < 0.01, ***p < 0.001.
(ZIP)

S2 Fig Effects of dopamine (DA) on mRNA and protein expression of factors in the YAP-TGF-β-Smad signaling pathway in ARPE-19 cells. (A) RT-PCR was used to detect the mRNA expression of *DRD1*, *DRD2*, *YAP*, *TEAD*, and *TGF-β2* in ARPE-19 cells, (B)Western blotting was used to detect the protein expression of *SMAD7, YAP, TEAD*, and *TGF-β2* in ARPE-19 cells, (C) Quantitative analysis of *DRD1, DRD2, YAP, TEAD* and *TGF-β2* mRNA expression levels in ARPE-19 cells.(D) quantitative results of protein expression of *SMAD7, YAP, TEAD,* and *TGF-β2* in ARPE-19 cells. (E) Protein expression of *TGF-β2* in the supernatant of ARPE-19 cell cultures, determined using ELISA. Data are reported as the means ± SD, n = 3. *p < 0.05, **p < 0.01, ***p < 0.001.
(ZIP)

S3 Fig ARPE-19 cell viability and migration ability after SCH23390 administration. (A–D) ARPE-19 cell viability after treatment with different concentrations of SCH23390 (12, 24, 48, or 92 μg/mL) for 6, 12, 24, or 48 h. The control group was treated similarly without the addition of SCH23390. (E) Transwell migration images of ARPE-19 cells treated with 0, 12, or 24 μg/mL SCH23390 for 0 and 12 h, and (F) the quantitative results. Scale bars: 100 μm. Data are reported as the means ± SD, n = 3. *p < 0.05, **p < 0.01, ***p < 0.001.
(ZIP)

S4 Fig Effects of SCH23390 on mRNA and protein expression of factors in the YAP-TGF-β-Smad signaling pathway in ARPE-19 cells. (A) RT-PCR was used to detect the mRNA expression of *DRD1*, *DRD2*, *YAP*, *TEAD*, and *TGF-β2* in ARPE-19 cells, (B)Western blotting was used to detect the protein expression of *SMAD7, YAP, TEAD*, and *TGF-β2* in ARPE-19 cells, (C)Quantitative analysis of *DRD1, DRD2, YAP, TEAD* and *TGF-β2* mRNA expression levels in ARPE-19 cells.(D) quantitative results of protein expression of *SMAD7, YAP, TEAD,* and *TGF-β2* in ARPE-19 cells. (E) Protein expression of *TGF-β2* in the supernatant of ARPE-19 cell cultures, determined using ELISA. Data are reported as the means ± SD, n = 3. *p < 0.05, **p < 0.01, ***p < 0.001.
(ZIP)

S5 Fig ARPE-19 cell viability and migration ability after sulpiride administration. (A–D) ARPE-19 cell viability after treatment with different concentrations of sulpiride (7, 14, 28, or 56 μg/mL) for 6, 12, 24, or 48 h. The control group was maintained under the same conditions without the addition of sulpiride. (E) Transwell migration images of ARPE-19 cells treated with 0, 7, or 14 μg/mL sulpiride for 0 and 12 h, and (F) the quantitative results. Scale bars: 100 μm. Data are reported as the means ± SD, n = 3. *p < 0.05, **p < 0.01, *** *p* < 0.001.
(ZIP)

S6 Fig Effects of sulpiride on mRNA and protein expression of factors in the YAP-TGF-β-Smad signaling pathway in ARPE-19 cells. (A) RT-PCR was used to detect the mRNA expression of *DRD1*, *DRD2*, *YAP*, *TEAD*, and *TGF-β2* in ARPE-19 cells, (B)Western blotting was used to detect the protein expression of *SMAD7, YAP, TEAD*, and *TGF-β2* in ARPE-19 cells, (C) Quantitative analysis of *DRD1, DRD2, YAP, TEAD* and *TGF-β2* mRNA expression levels in ARPE-19 cells.(D) quantitative results of protein expression of *SMAD7, YAP, TEAD,* and *TGF-β2* in ARPE-19 cells. (E) Protein expression of *TGF-β2* in the supernatant of ARPE-19 cell cultures, determined using ELISA. Data are reported as the means ± SD, n = 3. *p < 0.05, **p < 0.01, ***p < 0.001.
(ZIP)

S1 Appendix A Fig. The expression of various factors is altered after drug intervention in RPE cells, affecting sclera remodeling. (A) Dopamine (DA) treatment causes changes in the expression of several factors. (B) D1 receptor antagonist and D2 receptor antagonist treatment causes changes in the expression of relevant factors in cells.
(PDF)
