# Supplementary material for: Effect of dopamine on TGF-β2 secretion by human retinal pigment epithelial cells and the underlying mechanism
Source: PLoS One. 2025 Nov 4;20(11):e0335526. doi: 10.1371/journal.pone.0335526 (PMC12585080; doi:10.1371/journal.pone.0335526)
Supplement: S1 Fig — (A–D) ARPE-19 cell viability after treatment with different concentrations of DA (10, 20, 40, and 80 μg/mL) for 6, 12, 24, or 48 h. The control group was kept under the same conditions without adding DA. (E) Transwell migration images of ARPE-19 cells treated with 0, 10, or 20 μg/mL DA for 0 and 12 h, and (F) quantitative results. Scale bars: 100 μm. Data are reported as the means ± SD, n = 3. *p < 0.05, **p < 0.01, ***p < 0.001. (ZIP) [file pone.0335526.s001.zip › S1 Fig.zip/S1 FigABCD.pdf.pdf]

6h	浓度梯度	生存率1	生存率2	生存率3	生存率4	生存率5
	0	0.943400025	1.155799985	1.052000046	1.773399949	1.780500054
	10	2.063299894	2.075900078	1.873800039	2.021500111	2.050899982
	20	2.341300011	2.317600012	2.164200068	2.217099905	2.250200033
	40	1.925099969	2.109200001	2.081199884	1.995399952	2.083600044
	80	2.049999952	2.059299946	1.867499948	1.726199985	1.666300058

12h	浓度梯度	生存率1	生存率2	生存率3	生存率4	生存率5
	0	1.14260006	1.236900002	1.61500001	1.860999942	1.747900009
	10	2.063299894	1.873800039	1.825099945	2.021500111	2.050899982
	20	2.057100058	2.447299957	2.490700006	2.45810008	2.644799948
	40	2.336699963	2.185199976	2.097599983	2.149499893	2.501300097
	80	2.160000086	2.036600113	1.831900001	1.872499943	2.15260005

24h	浓度梯度	生存率1	生存率2	生存率3	生存率4	生存率5
	0	1.145799994	1.420600057	0.9921	1.336599946	1.343140006
	10	1.311100006	1.274499893	1.130500078	1.903800011	1.518359995
	20	2.557600021	2.48119998	2.286299944	2.141400099	2.182600021
	40	1.975000024	1.550400019	1.572399974	2.005199909	1.842700005
	80	1.201200008	1.244799972	1.258100033	1.290699959	1.308399987

48h	浓度梯度	生存率1	生存率2	生存率3	生存率4	生存率5
	0	1.526100039	1.872699976	2.106400013	2.495899916	2.156199932
	10	2.159199953	2.191600084	2.251100063	2.779000044	2.762300014
	20	2.46329999	2.514400005	2.606400013	2.860100031	2.807199955
	40	2.23119998	1.671300054	2.498100042	2.355900049	2.539400101
	80	1.849599957	1.843899965	1.941599965	2.097300053	2.182600021
