# Supplementary material for: Effect of dopamine on TGF-β2 secretion by human retinal pigment epithelial cells and the underlying mechanism
Source: PLoS One. 2025 Nov 4;20(11):e0335526. doi: 10.1371/journal.pone.0335526 (PMC12585080; doi:10.1371/journal.pone.0335526)
Supplement: S1 Fig — (A–D) ARPE-19 cell viability after treatment with different concentrations of DA (10, 20, 40, and 80 μg/mL) for 6, 12, 24, or 48 h. The control group was kept under the same conditions without adding DA. (E) Transwell migration images of ARPE-19 cells treated with 0, 10, or 20 μg/mL DA for 0 and 12 h, and (F) quantitative results. Scale bars: 100 μm. Data are reported as the means ± SD, n = 3. *p < 0.05, **p < 0.01, ***p < 0.001. (ZIP) [file pone.0335526.s001.zip › S1 Fig.zip/S1 FigF.pdf.pdf]

0 10 20

31.41551	32.94905	20.13936	66.61295	61.50966	57.42866	83.59047	77.1951	85.37729
