# Supplementary material for: Effect of dopamine on TGF-β2 secretion by human retinal pigment epithelial cells and the underlying mechanism
Source: PLoS One. 2025 Nov 4;20(11):e0335526. doi: 10.1371/journal.pone.0335526 (PMC12585080; doi:10.1371/journal.pone.0335526)
Supplement: S2 Fig — (A) RT-PCR was used to detect the mRNA expression of DRD1, DRD2, YAP, TEAD, and TGF-β2 in ARPE-19 cells, (B)Western blotting was used to detect the protein expression of SMAD7, YAP, TEAD, and TGF-β2 in ARPE-19 cells, (C) Quantitative analysis of DRD1, DRD2, YAP, TEAD and TGF-β2 mRNA expression levels in ARPE-19 cells.(D) quantitative results of protein expression of SMAD7, YAP, TEAD, and TGF-β2 in ARPE-19 cells. (E) Protein expression of TGF-β2 in the supernatant of ARPE-19 cell cultures, determined using ELISA. Data are reported as the means ± SD, n = 3. *p < 0.05, **p < 0.01, ***p < 0.001. (ZIP) [file pone.0335526.s002.zip › S2 Fig.zip/S2 FigC.pdf.pdf]

		0			10			20
DRD1	100	100	100	120.3257	120.0033	127.2244	142.1301	153.3602
DRD2	100	100	100	183.2071	199.5122	141.8062	241.4921	252.3578
TGF-β2	100	100	100	89.92318	104.6368	97.84934	71.15292	67.69532
YAP	100	100	100	244.0583	390.888	239.4805	512.0574	457.6774
TEAD	100	100	100	148.6553	125.1795	162.6131	156.7018	131.7301

154.9939
188.924
86.6099
551.3821
177.1668
