# Supplementary material for: Effect of dopamine on TGF-β2 secretion by human retinal pigment epithelial cells and the underlying mechanism
Source: PLoS One. 2025 Nov 4;20(11):e0335526. doi: 10.1371/journal.pone.0335526 (PMC12585080; doi:10.1371/journal.pone.0335526)
Supplement: S2 Fig — (A) RT-PCR was used to detect the mRNA expression of DRD1, DRD2, YAP, TEAD, and TGF-β2 in ARPE-19 cells, (B)Western blotting was used to detect the protein expression of SMAD7, YAP, TEAD, and TGF-β2 in ARPE-19 cells, (C) Quantitative analysis of DRD1, DRD2, YAP, TEAD and TGF-β2 mRNA expression levels in ARPE-19 cells.(D) quantitative results of protein expression of SMAD7, YAP, TEAD, and TGF-β2 in ARPE-19 cells. (E) Protein expression of TGF-β2 in the supernatant of ARPE-19 cell cultures, determined using ELISA. Data are reported as the means ± SD, n = 3. *p < 0.05, **p < 0.01, ***p < 0.001. (ZIP) [file pone.0335526.s002.zip › S2 Fig.zip/S2 FigD.pdf.pdf]

		0			10			20
TGF-β2	100	100	100	91.22743	84.24939	91.81468	66.37656	46.90548
YAP	100	100	100	113.6269	109.2783	122.119	126.7054	186.6432
TEAD	100	100	100	122.119	113.0788	116.6053	218.7353	165.9902
SMAD7	100	100	100	119.6633	109.3789	143.0526	197.5104	198.0038

72.39367
213.6156
234.0599
253.3267
