# Supplementary material for: Effect of dopamine on TGF-β2 secretion by human retinal pigment epithelial cells and the underlying mechanism
Source: PLoS One. 2025 Nov 4;20(11):e0335526. doi: 10.1371/journal.pone.0335526 (PMC12585080; doi:10.1371/journal.pone.0335526)
Supplement: S2 Fig — (A) RT-PCR was used to detect the mRNA expression of DRD1, DRD2, YAP, TEAD, and TGF-β2 in ARPE-19 cells, (B)Western blotting was used to detect the protein expression of SMAD7, YAP, TEAD, and TGF-β2 in ARPE-19 cells, (C) Quantitative analysis of DRD1, DRD2, YAP, TEAD and TGF-β2 mRNA expression levels in ARPE-19 cells.(D) quantitative results of protein expression of SMAD7, YAP, TEAD, and TGF-β2 in ARPE-19 cells. (E) Protein expression of TGF-β2 in the supernatant of ARPE-19 cell cultures, determined using ELISA. Data are reported as the means ± SD, n = 3. *p < 0.05, **p < 0.01, ***p < 0.001. (ZIP) [file pone.0335526.s002.zip › S2 Fig.zip/S2 FigE.pdf.pdf]

DA.0		0.082000002	0.006724	145.3372978
		0.0832	0.00692224	146.467192
		0.084299996	0.007106489	147.5035244
	10	0.073299997	0.005372889	137.1659001
		0.073100001	0.00534361	136.9784765
		0.0748	0.00559504	138.5722115
	20	0.064300001	0.00413449	128.7503853
		0.065499999	0.00429025	129.8702406
		0.066600002	0.00443556	130.8973777
