# Supplementary figures and images for: Effect of dopamine on TGF-β2 secretion by human retinal pigment epithelial cells and the underlying mechanism
Source: PLoS One. 2025 Nov 4;20(11):e0335526. doi: 10.1371/journal.pone.0335526 (PMC12585080; doi:10.1371/journal.pone.0335526)

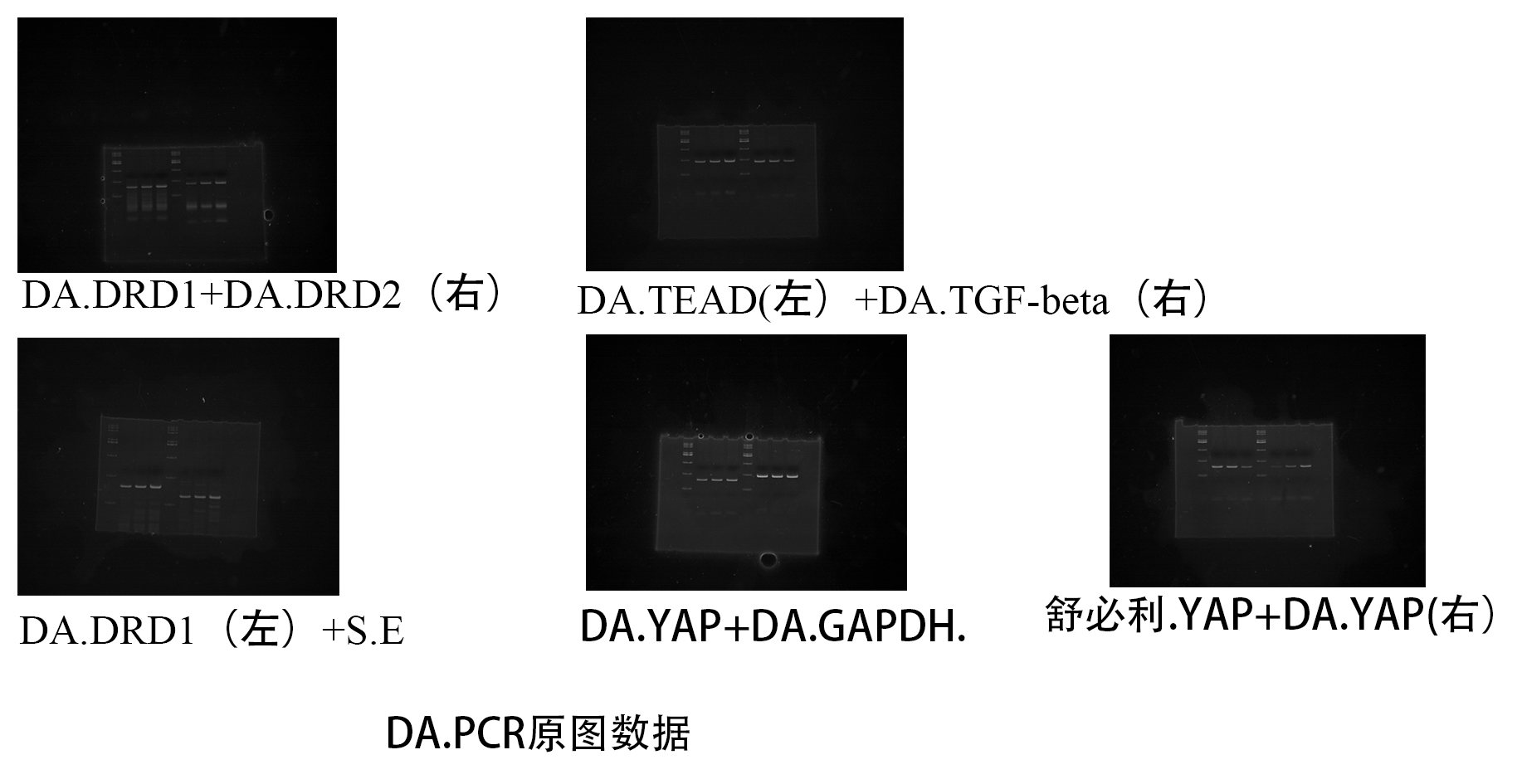

Supplement: S2 Fig — (A) RT-PCR was used to detect the mRNA expression of DRD1, DRD2, YAP, TEAD, and TGF-β2 in ARPE-19 cells, (B)Western blotting was used to detect the protein expression of SMAD7, YAP, TEAD, and TGF-β2 in ARPE-19 cells, (C) Quantitative analysis of DRD1, DRD2, YAP, TEAD and TGF-β2 mRNA expression levels in ARPE-19 cells.(D) quantitative results of protein expression of SMAD7, YAP, TEAD, and TGF-β2 in ARPE-19 cells. (E) Protein expression of TGF-β2 in the supernatant of ARPE-19 cell cultures, determined using ELISA. Data are reported as the means ± SD, n = 3. *p < 0.05, **p < 0.01, ***p < 0.001. (ZIP) [file pone.0335526.s002.zip › S2 Fig.zip/S2 FigA.pdf.tif]

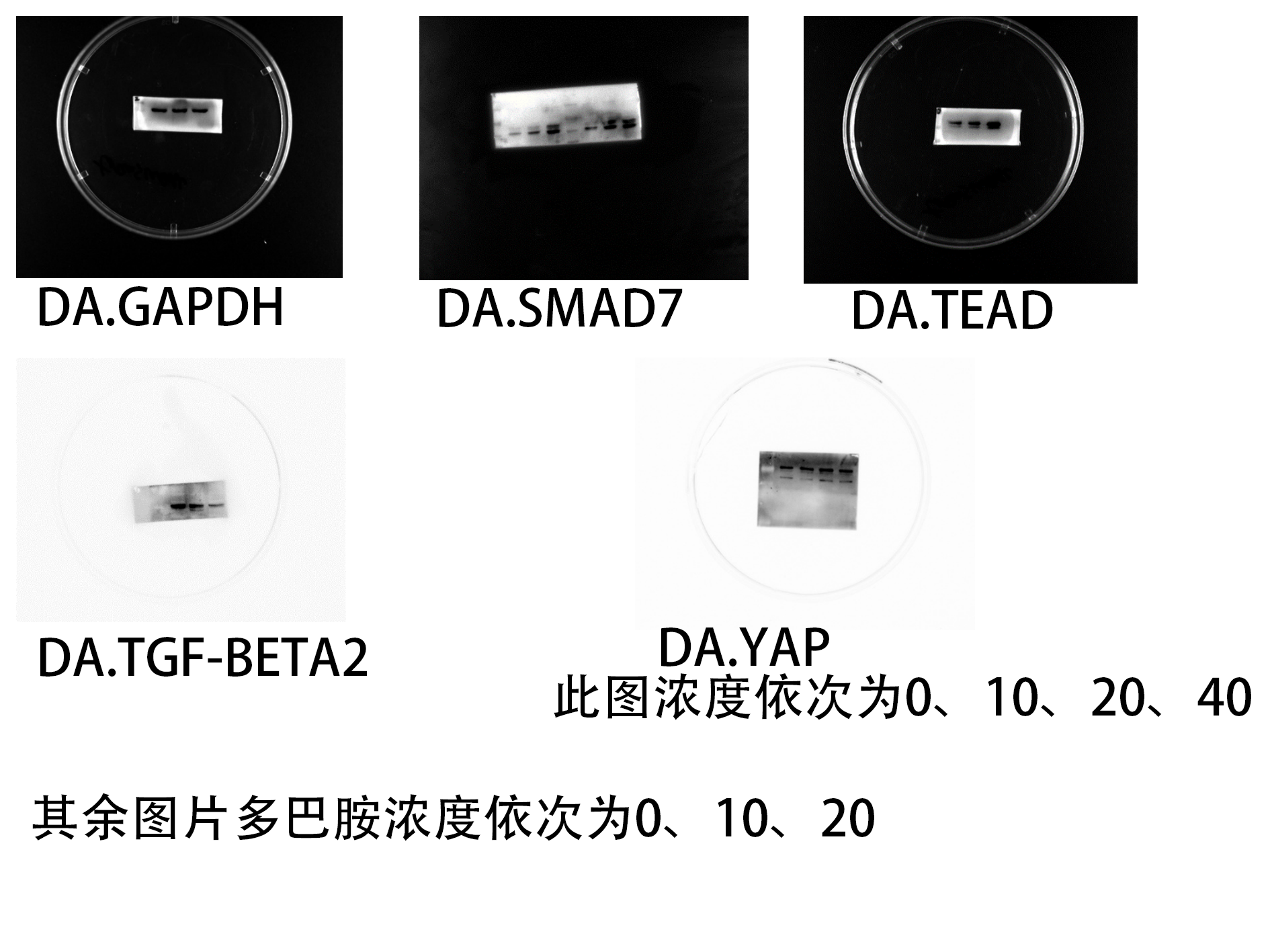

Supplement: S2 Fig — (A) RT-PCR was used to detect the mRNA expression of DRD1, DRD2, YAP, TEAD, and TGF-β2 in ARPE-19 cells, (B)Western blotting was used to detect the protein expression of SMAD7, YAP, TEAD, and TGF-β2 in ARPE-19 cells, (C) Quantitative analysis of DRD1, DRD2, YAP, TEAD and TGF-β2 mRNA expression levels in ARPE-19 cells.(D) quantitative results of protein expression of SMAD7, YAP, TEAD, and TGF-β2 in ARPE-19 cells. (E) Protein expression of TGF-β2 in the supernatant of ARPE-19 cell cultures, determined using ELISA. Data are reported as the means ± SD, n = 3. *p < 0.05, **p < 0.01, ***p < 0.001. (ZIP) [file pone.0335526.s002.zip › S2 Fig.zip/S2 FigB.pdf.tif]

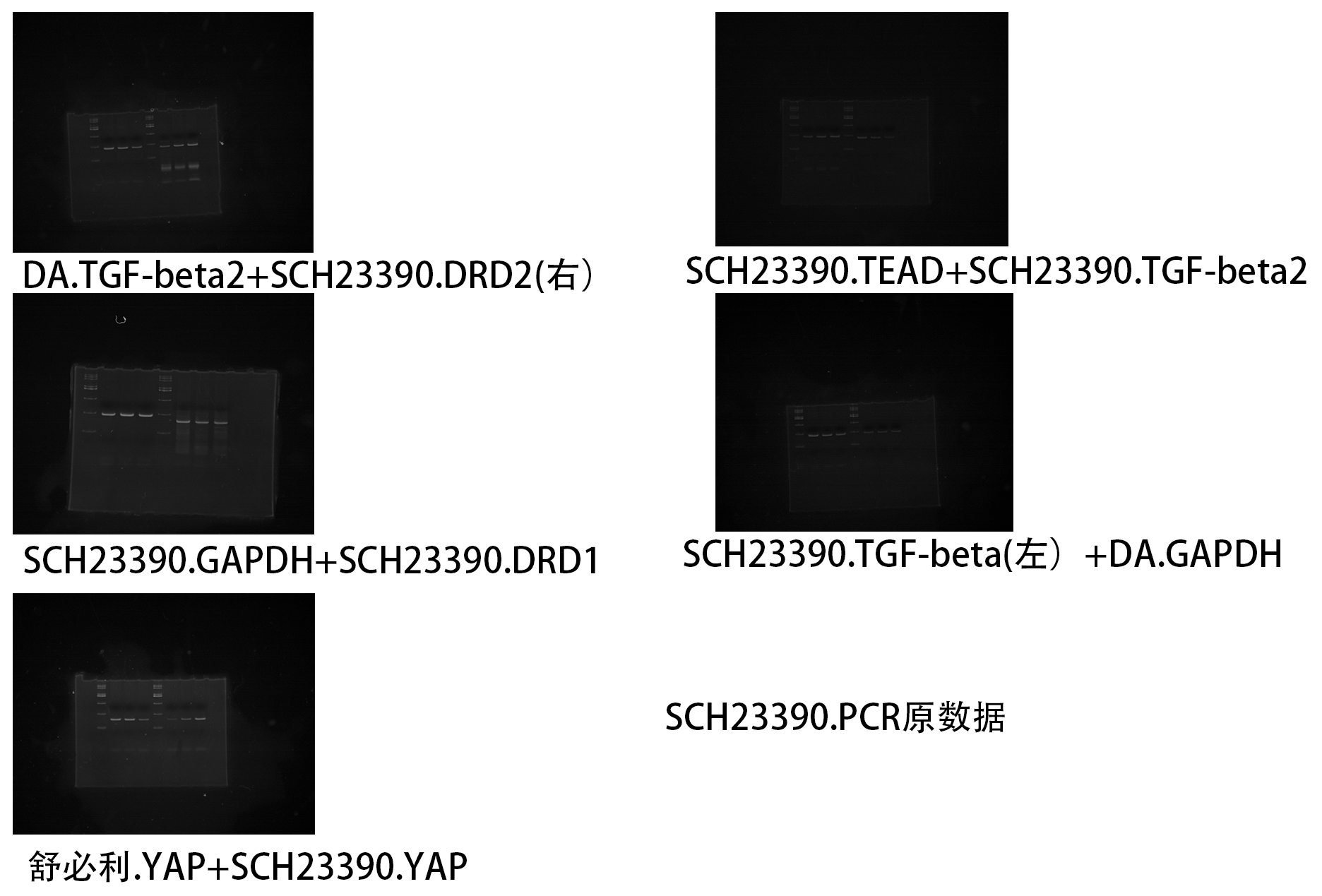

Supplement: S4 Fig — (A) RT-PCR was used to detect the mRNA expression of DRD1, DRD2, YAP, TEAD, and TGF-β2 in ARPE-19 cells, (B)Western blotting was used to detect the protein expression of SMAD7, YAP, TEAD, and TGF-β2 in ARPE-19 cells, (C)Quantitative analysis of DRD1, DRD2, YAP, TEAD and TGF-β2 mRNA expression levels in ARPE-19 cells.(D) quantitative results of protein expression of SMAD7, YAP, TEAD, and TGF-β2 in ARPE-19 cells. (E) Protein expression of TGF-β2 in the supernatant of ARPE-19 cell cultures, determined using ELISA. Data are reported as the means ± SD, n = 3. *p < 0.05, **p < 0.01, ***p < 0.001. (ZIP) [file pone.0335526.s004.zip › S4 Fig.zip/S4 FigA.pdf.tif]

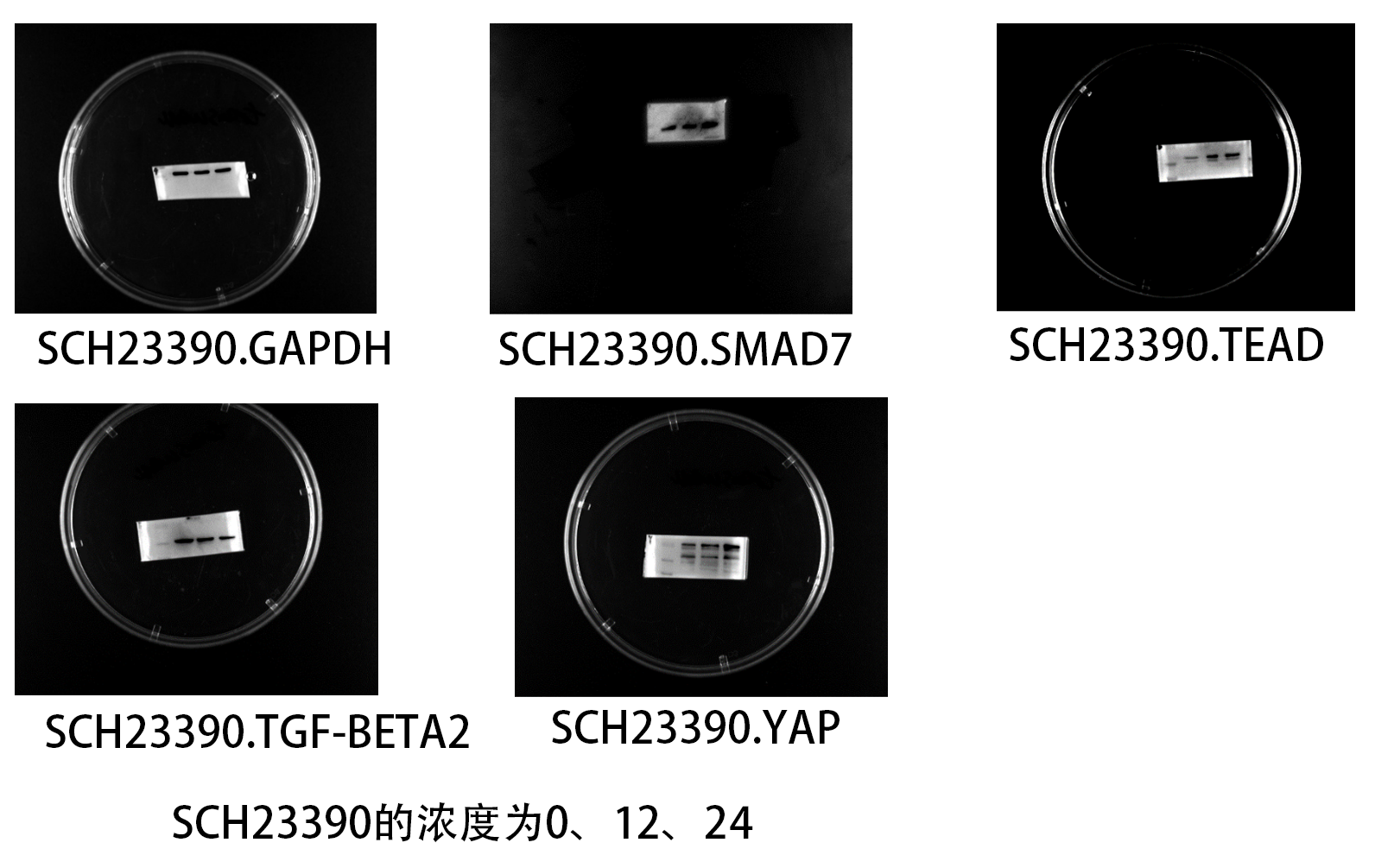

Supplement: S4 Fig — (A) RT-PCR was used to detect the mRNA expression of DRD1, DRD2, YAP, TEAD, and TGF-β2 in ARPE-19 cells, (B)Western blotting was used to detect the protein expression of SMAD7, YAP, TEAD, and TGF-β2 in ARPE-19 cells, (C)Quantitative analysis of DRD1, DRD2, YAP, TEAD and TGF-β2 mRNA expression levels in ARPE-19 cells.(D) quantitative results of protein expression of SMAD7, YAP, TEAD, and TGF-β2 in ARPE-19 cells. (E) Protein expression of TGF-β2 in the supernatant of ARPE-19 cell cultures, determined using ELISA. Data are reported as the means ± SD, n = 3. *p < 0.05, **p < 0.01, ***p < 0.001. (ZIP) [file pone.0335526.s004.zip › S4 Fig.zip/S4 FigB.pdf.tif]

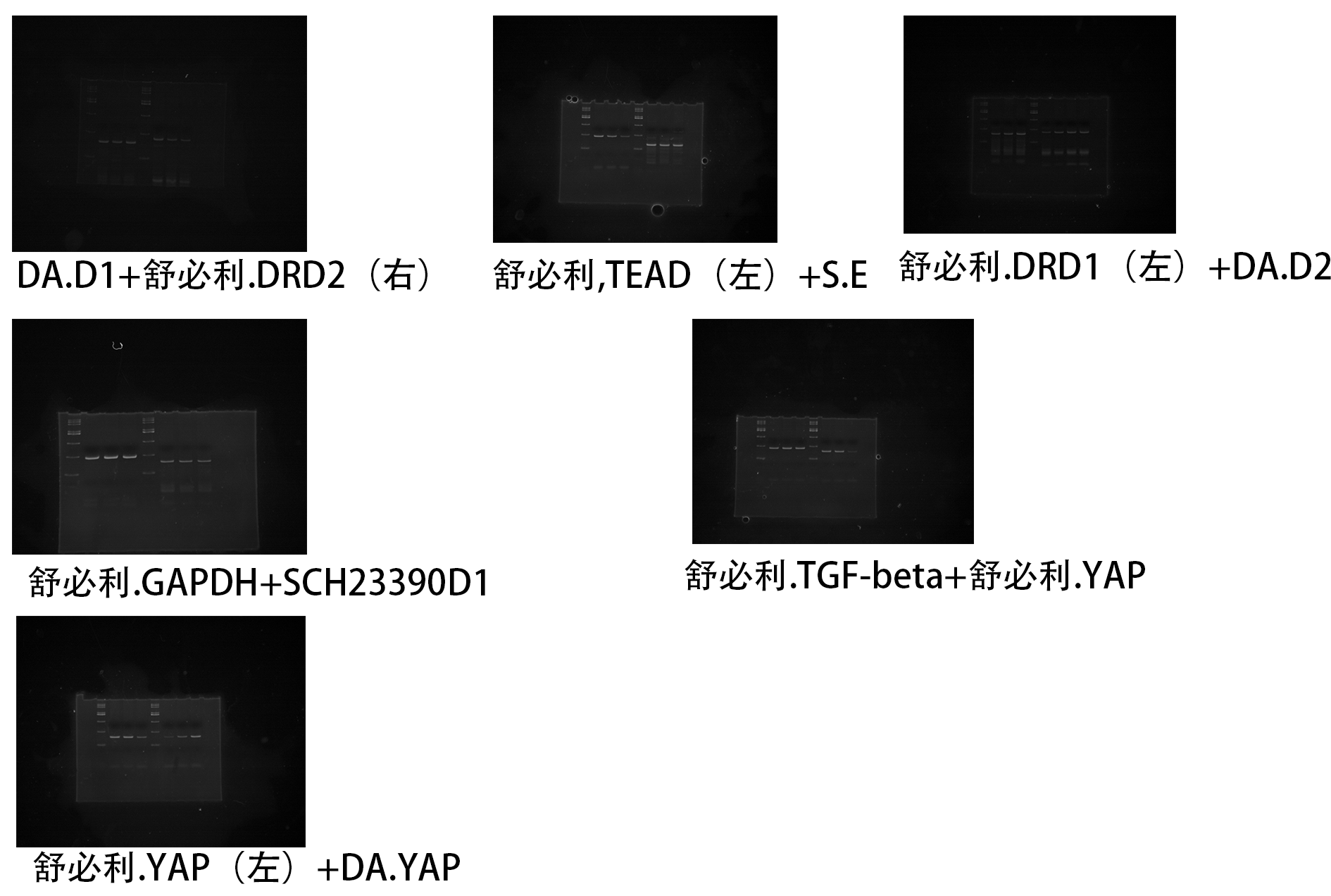

Supplement: S6 Fig — (A) RT-PCR was used to detect the mRNA expression of DRD1, DRD2, YAP, TEAD, and TGF-β2 in ARPE-19 cells, (B)Western blotting was used to detect the protein expression of SMAD7, YAP, TEAD, and TGF-β2 in ARPE-19 cells, (C) Quantitative analysis of DRD1, DRD2, YAP, TEAD and TGF-β2 mRNA expression levels in ARPE-19 cells.(D) quantitative results of protein expression of SMAD7, YAP, TEAD, and TGF-β2 in ARPE-19 cells. (E) Protein expression of TGF-β2 in the supernatant of ARPE-19 cell cultures, determined using ELISA. Data are reported as the means ± SD, n = 3. *p < 0.05, **p < 0.01, ***p < 0.001. (ZIP) [file pone.0335526.s006.zip › S6 Fig.zip/S6 FigA.pdf.tif]

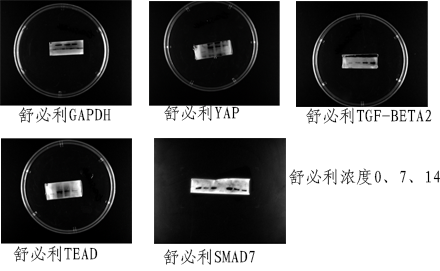

Supplement: S6 Fig — (A) RT-PCR was used to detect the mRNA expression of DRD1, DRD2, YAP, TEAD, and TGF-β2 in ARPE-19 cells, (B)Western blotting was used to detect the protein expression of SMAD7, YAP, TEAD, and TGF-β2 in ARPE-19 cells, (C) Quantitative analysis of DRD1, DRD2, YAP, TEAD and TGF-β2 mRNA expression levels in ARPE-19 cells.(D) quantitative results of protein expression of SMAD7, YAP, TEAD, and TGF-β2 in ARPE-19 cells. (E) Protein expression of TGF-β2 in the supernatant of ARPE-19 cell cultures, determined using ELISA. Data are reported as the means ± SD, n = 3. *p < 0.05, **p < 0.01, ***p < 0.001. (ZIP) [file pone.0335526.s006.zip › S6 Fig.zip/S6 FigB.pdf.tif]

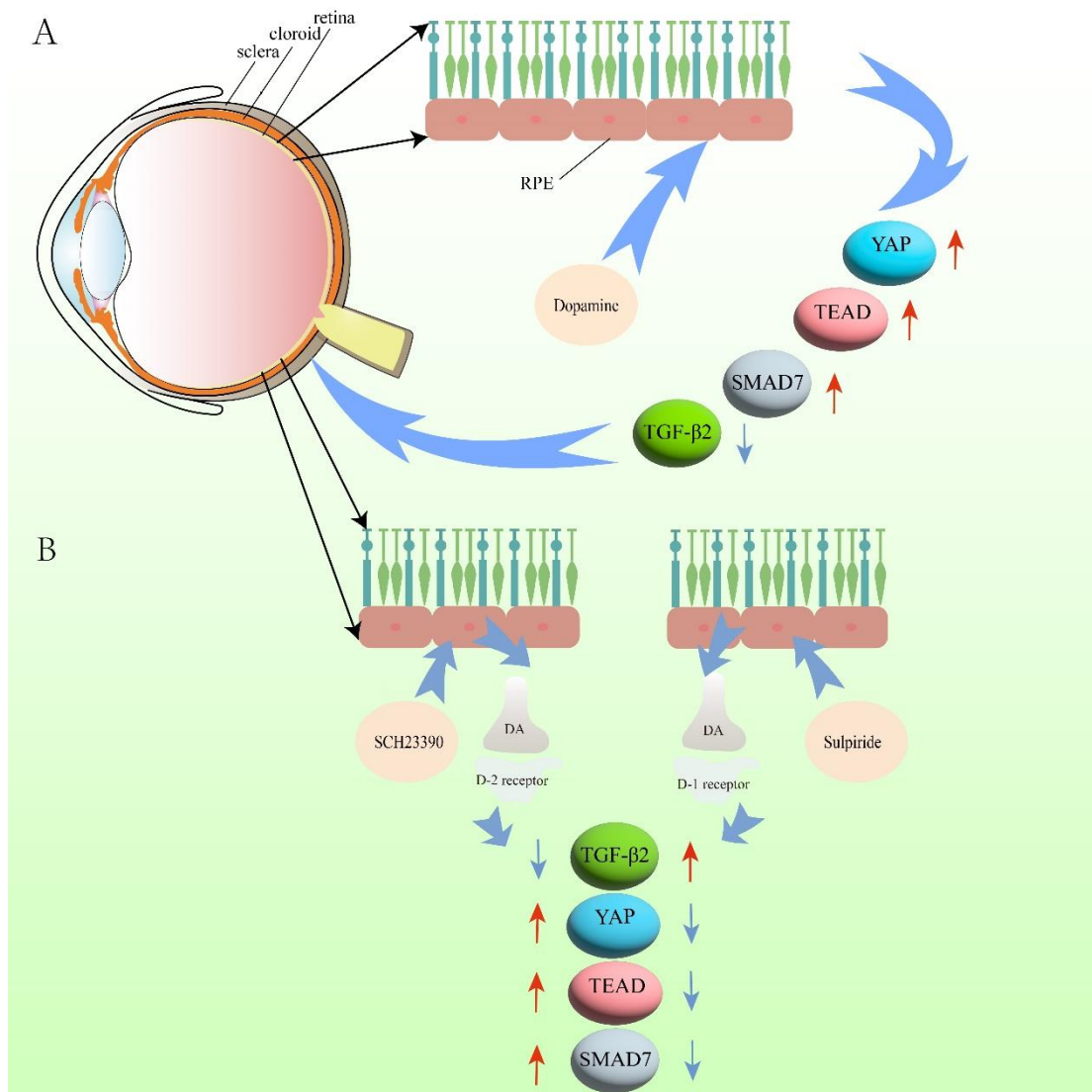

Supplement: S1 Appendix — (A) Dopamine (DA) treatment causes changes in the expression of several factors. (B) D1 receptor antagonist and D2 receptor antagonist treatment causes changes in the expression of relevant factors in cells. (PDF) [file pone.0335526.s007.pdf]
