# Supplementary material for: Effect of dopamine on TGF-β2 secretion by human retinal pigment epithelial cells and the underlying mechanism
Source: PLoS One. 2025 Nov 4;20(11):e0335526. doi: 10.1371/journal.pone.0335526 (PMC12585080; doi:10.1371/journal.pone.0335526)
Supplement: S3 Fig — (A–D) ARPE-19 cell viability after treatment with different concentrations of SCH23390 (12, 24, 48, or 92 μg/mL) for 6, 12, 24, or 48 h. The control group was treated similarly without the addition of SCH23390. (E) Transwell migration images of ARPE-19 cells treated with 0, 12, or 24 μg/mL SCH23390 for 0 and 12 h, and (F) the quantitative results. Scale bars: 100 μm. Data are reported as the means ± SD, n = 3. *p < 0.05, **p < 0.01, ***p < 0.001. (ZIP) [file pone.0335526.s003.zip › S3 Fig.zip/S3 FigABCD.pdf.pdf]

DRD1：6h

浓度梯度	生存率1	生存率2	生存率3	生存率4	生存率5
0	0.57160002	0.584500015	0.574299991	0.762399971	0.870999992
12	0.767700016	0.661599994	0.769200027	0.791899979	0.848500013
24	1.019999981	0.881500006	0.809599996	0.886799991	1.156499982
48	0.977999985	0.920000017	0.819999993	0.841899991	0.841099977
92	0.873000026	0.839600027	0.74059999	0.594200015	0.576499999

DRD1：12h

浓度梯度	生存率1	生存率2	生存率3	生存率4	生存率5
0	1.14260006	1.441100001	1.41900003	1.345399976	1.26970005
12	1.001000047	1.130599976	1.202	1.201900005	1.156499982
24	1.021199942	1.129500031	1.157799959	1.163599968	0.907500029
48	0.709200025	0.502600014	0.698099971	0.78670001	0.836000025
92	0.483500004	0.513599992	0.656499982	0.716700017	0.632099986

DRD1：24h

浓度梯度	生存率1	生存率2	生存率3	生存率4	生存率5
0	0.894299984	0.861899972	1.011899948	1.001000047	0.944800019
12	1.077000022	0.772599995	0.734700024	0.7324	0.744499981
24	1.047500014	0.622300029	0.714999974	0.729099989	0.819800019
48	0.913299978	0.511600018	0.770799994	0.646899998	0.716199994
92	0.680499971	0.67809999	0.680899978	0.598100007	0.339899987

DRD1：48h

浓度梯度	生存率1	生存率2	生存率3	生存率4	生存率5
0	2.471299887	2.444000006	2.781100035	3.853600025	2.404299974
12	2.276599884	1.096899986	1.591099977	1.831799984	1.773000002
24	1.333299994	1.63380003	1.889899969	1.636500001	1.450600028
40	0.71	0.707499981	1.121799946	1.220299959	1.180699944
92	0.466500014	0.713199973	0.989600003	1.246999979	1.077200055

生存率6
0.82069999
0.952400029
1.165500045
0.782100022
0.70569998

生存率6
1.22179997
1.090899944
0.828299999
0.662199974
0.818899989

生存率6
0.870199978
0.765399992
0.704299986
0.470400006
0.434500009

生存率6
2.523699999
1.70539999
1.420099974
1.314200044
0.750800014
