# Supplementary material for: Effect of dopamine on TGF-β2 secretion by human retinal pigment epithelial cells and the underlying mechanism
Source: PLoS One. 2025 Nov 4;20(11):e0335526. doi: 10.1371/journal.pone.0335526 (PMC12585080; doi:10.1371/journal.pone.0335526)
Supplement: S3 Fig — (A–D) ARPE-19 cell viability after treatment with different concentrations of SCH23390 (12, 24, 48, or 92 μg/mL) for 6, 12, 24, or 48 h. The control group was treated similarly without the addition of SCH23390. (E) Transwell migration images of ARPE-19 cells treated with 0, 12, or 24 μg/mL SCH23390 for 0 and 12 h, and (F) the quantitative results. Scale bars: 100 μm. Data are reported as the means ± SD, n = 3. *p < 0.05, **p < 0.01, ***p < 0.001. (ZIP) [file pone.0335526.s003.zip › S3 Fig.zip/S3 FigF.pdf.pdf]

	0			12			24	
71.88582	64.4904	65.94716	57.44834	56.25535	37.28601	21.98134	16.90028	27.63468
