# Supplementary material for: Effect of dopamine on TGF-β2 secretion by human retinal pigment epithelial cells and the underlying mechanism
Source: PLoS One. 2025 Nov 4;20(11):e0335526. doi: 10.1371/journal.pone.0335526 (PMC12585080; doi:10.1371/journal.pone.0335526)
Supplement: S4 Fig — (A) RT-PCR was used to detect the mRNA expression of DRD1, DRD2, YAP, TEAD, and TGF-β2 in ARPE-19 cells, (B)Western blotting was used to detect the protein expression of SMAD7, YAP, TEAD, and TGF-β2 in ARPE-19 cells, (C)Quantitative analysis of DRD1, DRD2, YAP, TEAD and TGF-β2 mRNA expression levels in ARPE-19 cells.(D) quantitative results of protein expression of SMAD7, YAP, TEAD, and TGF-β2 in ARPE-19 cells. (E) Protein expression of TGF-β2 in the supernatant of ARPE-19 cell cultures, determined using ELISA. Data are reported as the means ± SD, n = 3. *p < 0.05, **p < 0.01, ***p < 0.001. (ZIP) [file pone.0335526.s004.zip › S4 Fig.zip/S4 FigC.pdf.pdf]

	0			12			24	
DRD1	100	100	100	70.23318	85.40625	84.21361	59.34045	77.31713
DRD2	100	100	100	101.6371	176.8646	178.4896	117.5941	264.6861
TGF-β2	100	100	100	85.86393	89.9226	92.43051	71.85452	33.09409
YAP	100	100	100	227.9711	118.2188	224.988	413.4826	369.5306
TEAD	100	100	100	118.8909	126.692	134.3654	121.6767	132.359

79.34914
241.9427
77.11411
370.8351
151.9553
