# Supplementary material for: Effect of dopamine on TGF-β2 secretion by human retinal pigment epithelial cells and the underlying mechanism
Source: PLoS One. 2025 Nov 4;20(11):e0335526. doi: 10.1371/journal.pone.0335526 (PMC12585080; doi:10.1371/journal.pone.0335526)
Supplement: S4 Fig — (A) RT-PCR was used to detect the mRNA expression of DRD1, DRD2, YAP, TEAD, and TGF-β2 in ARPE-19 cells, (B)Western blotting was used to detect the protein expression of SMAD7, YAP, TEAD, and TGF-β2 in ARPE-19 cells, (C)Quantitative analysis of DRD1, DRD2, YAP, TEAD and TGF-β2 mRNA expression levels in ARPE-19 cells.(D) quantitative results of protein expression of SMAD7, YAP, TEAD, and TGF-β2 in ARPE-19 cells. (E) Protein expression of TGF-β2 in the supernatant of ARPE-19 cell cultures, determined using ELISA. Data are reported as the means ± SD, n = 3. *p < 0.05, **p < 0.01, ***p < 0.001. (ZIP) [file pone.0335526.s004.zip › S4 Fig.zip/S4 FigD.pdf.pdf]

		0			12			24
TGF-β2	100	100	100	99.15694	92.26244	91.22016	83.32205	80.57606
YAP	100	100	100	150.1126	105.1446	109.1379	183.9853	145.6708
TEAD	100	100	100	143.2792	150.0305	115.8284	227.6386	218.4172
SMAD7	100	100	100	110.6782	100.0429	100.5839	161.9469	153.9541

69.40437
172.8841
153.5329
159.2459
