# Supplementary material for: Effect of dopamine on TGF-β2 secretion by human retinal pigment epithelial cells and the underlying mechanism
Source: PLoS One. 2025 Nov 4;20(11):e0335526. doi: 10.1371/journal.pone.0335526 (PMC12585080; doi:10.1371/journal.pone.0335526)
Supplement: S4 Fig — (A) RT-PCR was used to detect the mRNA expression of DRD1, DRD2, YAP, TEAD, and TGF-β2 in ARPE-19 cells, (B)Western blotting was used to detect the protein expression of SMAD7, YAP, TEAD, and TGF-β2 in ARPE-19 cells, (C)Quantitative analysis of DRD1, DRD2, YAP, TEAD and TGF-β2 mRNA expression levels in ARPE-19 cells.(D) quantitative results of protein expression of SMAD7, YAP, TEAD, and TGF-β2 in ARPE-19 cells. (E) Protein expression of TGF-β2 in the supernatant of ARPE-19 cell cultures, determined using ELISA. Data are reported as the means ± SD, n = 3. *p < 0.05, **p < 0.01, ***p < 0.001. (ZIP) [file pone.0335526.s004.zip › S4 Fig.zip/S4 FigE.pdf.pdf]

S.0		0.066699997	0.00444889	130.9907765
		0.068899997	0.00474721	133.0468635
		0.069600001	0.00484416	133.7015568
	12	0.0744	0.00553536	138.1970925
		0.070799999	0.00501264	134.8244181
		0.0704	0.00495616	134.4500554
	24	0.082699999	0.00683929	145.9963183
		0.084100001	0.00707281	147.315061
		0.085900001	0.00737881	149.0119475
