# Supplementary material for: Effect of dopamine on TGF-β2 secretion by human retinal pigment epithelial cells and the underlying mechanism
Source: PLoS One. 2025 Nov 4;20(11):e0335526. doi: 10.1371/journal.pone.0335526 (PMC12585080; doi:10.1371/journal.pone.0335526)
Supplement: S5 Fig — (A–D) ARPE-19 cell viability after treatment with different concentrations of sulpiride (7, 14, 28, or 56 μg/mL) for 6, 12, 24, or 48 h. The control group was maintained under the same conditions without the addition of sulpiride. (E) Transwell migration images of ARPE-19 cells treated with 0, 7, or 14 μg/mL sulpiride for 0 and 12 h, and (F) the quantitative results. Scale bars: 100 μm. Data are reported as the means ± SD, n = 3. *p < 0.05, **p < 0.01, *** p < 0.001. (ZIP) [file pone.0335526.s005.zip › S5 Fig.zip/S5 FigABCD.pdf.pdf]

舒必利6h

浓度梯度	生存率1	生存率2	生存率3	生存率4	生存率5
0	0.626399994	0.862999976	0.846800029	0.77640003	0.834500015
10	0.829100013	0.801900029	0.813799977	0.943499982	0.885800004
20	0.822799981	0.824299991	0.905399978	0.870299995	0.905600011
40	0.773500025	0.789399981	0.892700016	0.867200017	0.750199974
80	0.555100024	0.888100028	0.672200024	0.834399998	0.892400026

舒必利12h

浓度梯度	生存率1	生存率2	生存率3	生存率4	生存率5
0	0.729499996	0.453599989	0.754199982	0.677200019	0.788999975
10	0.947000027	1.286900043	0.923500001	0.954400003	0.880800009
20	1.374500036	1.162899971	1.002400041	0.968900025	1.129400015
40	0.578199983	1.042999983	0.659300029	0.807600021	0.973100007
80	0.502600014	0.919499993	1.002200007	0.696500003	0.649299979

舒必利24h

浓度梯度	生存率1	生存率2	生存率3	生存率4	生存率5
0	0.502600014	0.919499993	1.002200007	0.696500003	0.649299979
10	1.006100059	0.729499996	0.754199982	0.677200019	0.788999975
20	1.516600013	1.374500036	1.162899971	1.002400041	0.968900025
40	0.947000027	1.286900043	1.129400015	0.954400003	0.880800009
80	0.578199983	1.042999983	0.659300029	0.807600021	0.973100007

舒必利48h

浓度梯度	生存率1	生存率2	生存率3	生存率4	生存率5
0	0.938799977	0.779799998	0.959200025	0.861000001	0.880699992
10	0.951799989	1.012500048	0.996200025	0.984399974	0.975700021
20	1.209400058	1.135200024	1.16170001	1.111199975	1.04460001
40	1.090399981	1.092800021	1.036800027	0.945800006	1.030599952
80	0.923200011	1.121500015	0.965699971	1.040500045	0.996500015
