# Supplementary material for: Effect of dopamine on TGF-β2 secretion by human retinal pigment epithelial cells and the underlying mechanism
Source: PLoS One. 2025 Nov 4;20(11):e0335526. doi: 10.1371/journal.pone.0335526 (PMC12585080; doi:10.1371/journal.pone.0335526)
Supplement: S5 Fig — (A–D) ARPE-19 cell viability after treatment with different concentrations of sulpiride (7, 14, 28, or 56 μg/mL) for 6, 12, 24, or 48 h. The control group was maintained under the same conditions without the addition of sulpiride. (E) Transwell migration images of ARPE-19 cells treated with 0, 7, or 14 μg/mL sulpiride for 0 and 12 h, and (F) the quantitative results. Scale bars: 100 μm. Data are reported as the means ± SD, n = 3. *p < 0.05, **p < 0.01, *** p < 0.001. (ZIP) [file pone.0335526.s005.zip › S5 Fig.zip/S5 FigF.pdf.pdf]

	0			7			14	
43.17557	34.54732	29.97897	51.93108	48.14649	35.06622	69.88669	70.77136	64.58517
