# Supplementary material for: Effect of dopamine on TGF-β2 secretion by human retinal pigment epithelial cells and the underlying mechanism
Source: PLoS One. 2025 Nov 4;20(11):e0335526. doi: 10.1371/journal.pone.0335526 (PMC12585080; doi:10.1371/journal.pone.0335526)
Supplement: S6 Fig — (A) RT-PCR was used to detect the mRNA expression of DRD1, DRD2, YAP, TEAD, and TGF-β2 in ARPE-19 cells, (B)Western blotting was used to detect the protein expression of SMAD7, YAP, TEAD, and TGF-β2 in ARPE-19 cells, (C) Quantitative analysis of DRD1, DRD2, YAP, TEAD and TGF-β2 mRNA expression levels in ARPE-19 cells.(D) quantitative results of protein expression of SMAD7, YAP, TEAD, and TGF-β2 in ARPE-19 cells. (E) Protein expression of TGF-β2 in the supernatant of ARPE-19 cell cultures, determined using ELISA. Data are reported as the means ± SD, n = 3. *p < 0.05, **p < 0.01, ***p < 0.001. (ZIP) [file pone.0335526.s006.zip › S6 Fig.zip/S6 FigC.pdf.pdf]

		0			7			14
DRD1	100	100	100	151.0189	125.3849	104.2684	181.4416	162.3437
DRD2	100	100	100	53.09272	55.57478	90.41031	24.14236	27.63801
TGF-β2	100	100	100	127.3857	103.0054	109.6541	162.639	113.5978
YAP	100	100	100	86.17537	85.11504	97.15246	41.08729	17.60265
TEAD	100	100	100	81.55044	71.3041	71.86443	74.04395	28.99833

110.9961
77.25442
126.4452
24.412
38.80877
