# Supplementary material for: Effect of dopamine on TGF-β2 secretion by human retinal pigment epithelial cells and the underlying mechanism
Source: PLoS One. 2025 Nov 4;20(11):e0335526. doi: 10.1371/journal.pone.0335526 (PMC12585080; doi:10.1371/journal.pone.0335526)
Supplement: S6 Fig — (A) RT-PCR was used to detect the mRNA expression of DRD1, DRD2, YAP, TEAD, and TGF-β2 in ARPE-19 cells, (B)Western blotting was used to detect the protein expression of SMAD7, YAP, TEAD, and TGF-β2 in ARPE-19 cells, (C) Quantitative analysis of DRD1, DRD2, YAP, TEAD and TGF-β2 mRNA expression levels in ARPE-19 cells.(D) quantitative results of protein expression of SMAD7, YAP, TEAD, and TGF-β2 in ARPE-19 cells. (E) Protein expression of TGF-β2 in the supernatant of ARPE-19 cell cultures, determined using ELISA. Data are reported as the means ± SD, n = 3. *p < 0.05, **p < 0.01, ***p < 0.001. (ZIP) [file pone.0335526.s006.zip › S6 Fig.zip/S6 FigD.pdf.pdf]

	0			7			14		
TGF-β2	100	100	100	110.2236	118.0887	133.2032	118.9291	125.1581	
YAP	100	100	100	72.93708	54.89518	99.74589	43.82715	34.3024	
TEAD	100	100	100	90.6323	65.74682	68.35239	61.13318	32.18268	
SMAD7	100	100	100	45.26674	77.63117	36.80356	34.34205	55.70411	

144.493
72.31203
37.1047
23.63235
