# Supplementary material for: Effect of dopamine on TGF-β2 secretion by human retinal pigment epithelial cells and the underlying mechanism
Source: PLoS One. 2025 Nov 4;20(11):e0335526. doi: 10.1371/journal.pone.0335526 (PMC12585080; doi:10.1371/journal.pone.0335526)
Supplement: S6 Fig — (A) RT-PCR was used to detect the mRNA expression of DRD1, DRD2, YAP, TEAD, and TGF-β2 in ARPE-19 cells, (B)Western blotting was used to detect the protein expression of SMAD7, YAP, TEAD, and TGF-β2 in ARPE-19 cells, (C) Quantitative analysis of DRD1, DRD2, YAP, TEAD and TGF-β2 mRNA expression levels in ARPE-19 cells.(D) quantitative results of protein expression of SMAD7, YAP, TEAD, and TGF-β2 in ARPE-19 cells. (E) Protein expression of TGF-β2 in the supernatant of ARPE-19 cell cultures, determined using ELISA. Data are reported as the means ± SD, n = 3. *p < 0.05, **p < 0.01, ***p < 0.001. (ZIP) [file pone.0335526.s006.zip › S6 Fig.zip/S6 FigE.pdf.pdf]

H.0		0.077600002	0.00602176	141.2001685
		0.0766	0.00586756	140.261186
		0.076399997	0.00583696	140.0734435
	7	0.074900001	0.00561001	138.6660048
		0.074000001	0.005476	137.8220492
		0.075999998	0.005776	139.698022
	14	0.067000002	0.004489	131.271022
		0.0682	0.00465124	132.3924087
		0.064199999	0.00412164	128.657093
